# Refractive surgery today: is there innovation or stagnation?

**DOI:** 10.1186/s40662-014-0004-0

**Published:** 2014-10-16

**Authors:** Jorge Alio

**Affiliations:** Ophthalmology, Miguel Hernandez University, Alicante, Spain; Vissum Corporation, Alicante, Spain

Refractive surgery, both corneal and intraocular, has undergone an explosive evolution during the last 25 years. The first excimer laser surgery performed now already over 25 years ago is a historical landmark which started the scientific age of corneal refractive surgery.

Before that momentous occasion, refractive surgery was a buccaneer procedure that lacked consistent scientific knowledge about the long-term outcomes of the procedures that were practiced (mainly radial and astigmatic keratotomy). In other words, there was a complete lack of objective outcome analysis coupled with almost no perspective on what the future development of this subspecialty was going to hold [1]. A classic example of this is Radial Keratotomy, which was extensively performed in Europe, the US and Latin America at the end of the 80’s and early 90’s. Only the PERK study was able to detect and announce the trend towards hyperopic flattening of these corneas years later when they approached the technique in a systematic way by studying a relatively small number of cases [2-4].

The introduction of excimer laser in the practice of refractive surgery was an exciting innovation. Large investments from the industry and even from doctors and hospitals created a huge demand for adequate information on outcomes as the possibilities of large-scale application of this technology were immediately seen. This challenge forced the companies involved in the ophthalmic development of the excimer laser technology to immediately perform clinical studies or refractive corneal surgery, which reached its peak in the first and second decades after its introduction in Europe. Almost everything that could be known about efficacy, predictability and stability, complications, technological development and innovation was then reported and accomplished in these two decades [5,6]. Currently, corneal refractive surgery performed by excimer laser has matured considerably and is able to cover from +6 to −12 diopters of sphere and up to 6 diopters of astigmatism with good, predictable and safe outcomes [7-10]. Hyperopia has been one of the frontiers approached with caution by modern corneal refractive surgeons, but recent evidence has confirmed its feasibility for up to 7 diopters with acceptable induction of HOA, equivalent to those induced by previous generations of excimer laser corneal surgical devices in hyperopic corrections up to +3 spherical diopters [10].

The development of intraocular phakic lenses followed a similar process [11]. The first phakic lenses were implanted in the 50’s with very doubtful outcomes, but it was in the late 80’s and early 90’s when this type of surgery was successfully accomplished in Europe. Years later, it was reported that these lenses could be anterior chamber angle- iris- and even lens-supported (retro-iridian). The indications and preliminary study of the patient has been enriched by advanced technologies such as anterior segment ocular coherence tomography (OCT), which makes the indications much more controllable. As is often the case with innovation, phakic intraocular lenses were initially considered with scepticism and criticized by many surgeons, only to be acknowledged later on as having a great potential in the correction of high refractive errors of all types [12,13].

In parallel, taking into account everything that has been mentioned above, major improvements in diagnostic equipment have provided refractive surgeons with a vast amount of information regarding the quality of refractive and optical conditions of the cornea using different technologies of corneal topography mapping and analysis, study of the optical properties of the eye by double pass and ray tracing analysis diagnostic imaging of the cornea such as the very high frequency ultrasound, laser interferometry, OCT, in vivo analysis of the different components of the corneal anatomy such as specular microscopy of the corneal endothelium and confocal microscopy, corneal biomechanical analysis and emerging techniques for the analysis of the tears and the condition of the ocular surface. All of these today give unique opportunities to better understand the condition of the eyes, which are candidates for refractive surgery, and the possible outcomes and consequences of the different refractive surgical procedures.

More recently, new corneal refractive surgical techniques are emerging with femtosecond corneal surgery being one of the most conspicuous and outstanding innovations in the field. At present, we can state that in myopic cases of up to 10 diopters with astigmatism up to 3, intrastromal femtosecond laser assisted lenticular corneal excision (Flex-Smile) is feasible and has a relatively similar predictability to excimer laser surgery with the potential advantages with respect to corneal stability [14-16]. If this technique is demonstrated to provide advantages over excimer laser surgery, femtosecond corneal surgery might find indications in larger refractive defects and in hyperopia. The future is open for these innovations as they seem to have potential advantages concerning the induction of postoperative corneal aberrations.

Other important examples of emerging refractive surgical techniques fall into the field of pseudophakic intraocular lenses. It has been well demonstrated that high hyperopic and myopic eyes have different optical profiles versus the normal emetropic eye [17]. Bearing this in mind, we can acknowledge that an adequately customized phakic intraocular lens may very well be a great benefit for the implanted eye if properly indicated seeing that we have access to so much information that allows us to customize such intraocular devices.

Future important areas of progress in modern refractive surgery will be based probably on the refractive use of modern corneoplastic techniques such as newly designed intraocular rings and segments, and corneal stiffening techniques such as collagen crosslinking, an emerging process that has just been explored.

Concerning the potential for visual improvement in handicapped eyes such as anisometropic amblyopia, possible future development of adaptive optic-based intraocular lenses may increase the visual potential of these eyes. This relatively new area of clinical research may have an enormous impact on the quality of life of visually handicapped patients and even normal patients who possibly could achieve the “super vision” paradigm that started, but failed when customized excimer laser surgery was introduced in early 2000 [18].

In summary, refractive surgery has not and will never reach stagnation as it affects the quality of vision and in turn, the quality of life for the average human being. The tremendous progress that has occurred over the last 25 years that I have witnessed is going to be followed by more sophisticated, highly scientific, and highly technologically developed refractive surgery procedures that will improve the quality of life of our patients. This will be done so by increasing the quality of vision and providing spectacle independence in a different way from the initial approaches performed over 25 years ago, and which started with so little knowledge. Never before in the history of ophthalmology has a subspecialty such as refractive surgery emerged from nothing to attain such a high level of expectation and innovation in technological advancement. As Arthur C. Clarke, the famous writer stated, “Any technology reaching very high levels of development might be indistinguishable from magic”. Today, the sophistication of refractive surgery is indeed reaching a level of magic from which only further improvements should be expected.
